# Control of the stochastic response of magnetization dynamics in spin-torque oscillator through radio-frequency magnetic fields

**DOI:** 10.1038/s41598-021-95636-w

**Published:** 2021-08-11

**Authors:** Sumito Tsunegi, Tomohiro Taniguchi, Daiki Suzuki, Kay Yakushiji, Akio Fukushima, Shinji Yuasa, Hitoshi Kubota

**Affiliations:** 1 National Institute of Advanced Industrial Science and Technology (AIST), Research Center for Emerging Computing Technologies, Tsukuba, 305–8568 Japan; 2 Japan Science and Technology Agency (JST), PRESTO, 4-1-8 Honcho, Kawaguchi, Saitama 332-0012 Japan

**Keywords:** Magnetic devices, Electronic and spintronic devices

## Abstract

Neuromorphic computing using spintronic devices, such as spin-torque oscillators (STOs), has been intensively studied for energy-efficient data processing. One of the critical issues in this application is stochasticity in magnetization dynamics, which limits the accuracy of computation. Such stochastic behavior, however, plays a key role in stochastic computing and machine learning. It is therefore important to develop methods for both suppressing and enhancing stochastic response in spintronic devices. We report on experimental investigations on control of stochastic quantity, such as the width of a distribution of transient time in magnetization dynamics in vortex-type STO. The spin-transfer effect can suppress stochasticity in transient dynamics from a non-oscillating to oscillating state, whereas an application of a radio-frequency magnetic field is effective in reducing stochasticity on the time evolution of the oscillating state.

Much recent works in spintronics have involved magnetoresistance and spin-transfer effects in ferromagnetic multilayers for practical applications such as magnetoresistive random access memory (MRAM)^1^ and spin-torque oscillators (STOs)^2^. One of the fascinating new research directions in spintronics is application of nonlinear^3–6^ and/or stochastic behaviors^7,8^ in nanomagnets to data processing such as neuromorphic computing and stochastic computing which aim to achieve energy efficient computations compared with conventional ones.

One of the critical issues in the new computing applications based on spintronic devices is stochasticity of magnetization dynamics due to thermal fluctuation^9–12^. It should be noted that conventional device applications, such as MRAM and STOs, use only the steady-state behaviors of magnetization, for example, magnetization switching to specific directions in MRAM and steady-state precession with unique frequency in STOs. On the other hand, the new computing applications such as physical reservoir computing use time-dependent transient response of nanomagnets in spintronics devices^3,13–20^. The thermal fluctuation in nanomagnets makes the dynamic response stochastic, which reduces the reproducibility of physical reservoir computing^16,17^. Although stochastic dynamics is harmful for physical reservoir computing, it is necessary for stochastic computing^21,22^. Stochasticity is also known to prevent falling into local minima in machine learning. Therefore, a method of controlling stochastic behaviors of transient phenomena in nanomagnets is necessary. However, only a few studies focused on the stochastic magnetization dynamics in transient phenomena in the time domain in detail^23–27^.

In this study, we experimentally investigated the stochastic transient response of an STO consisting of the magnetic vortex state because vortex-type STOs were used for physical reservoir computing in our previous study^3^. We measured the time-domain signal of the STO by applying pulse voltage and obtained the stochastic transient response. Stochasticity can be significantly suppressed by applying a radio frequency (RF) magnetic field to the ferromagnet.

## Results

### Evaluation of waiting time in transient dynamics

**Figure 1:**
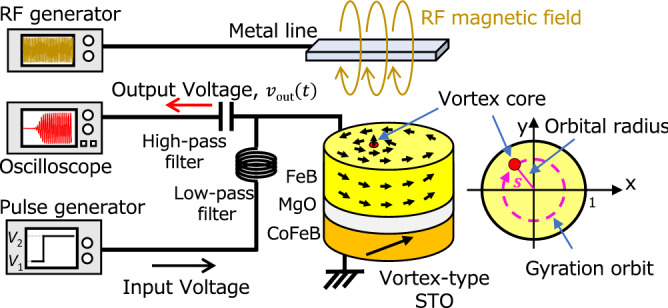
(**a**) Schematic of experimental setup for measuring transient behavior in vortex type spin-torque oscillator (STO) and its top view. Input voltage pulse was generated from pulse generator and injected into STO through low-pass filter. Output waveform was measured using oscilloscope through high-pass filter. Radio frequency (RF) magnetic field from metal line was applied to STO in experiment discussed the evaluation of $t_{{{\text{wait}}}}^{^{\prime}}$.

**Figure 2:**
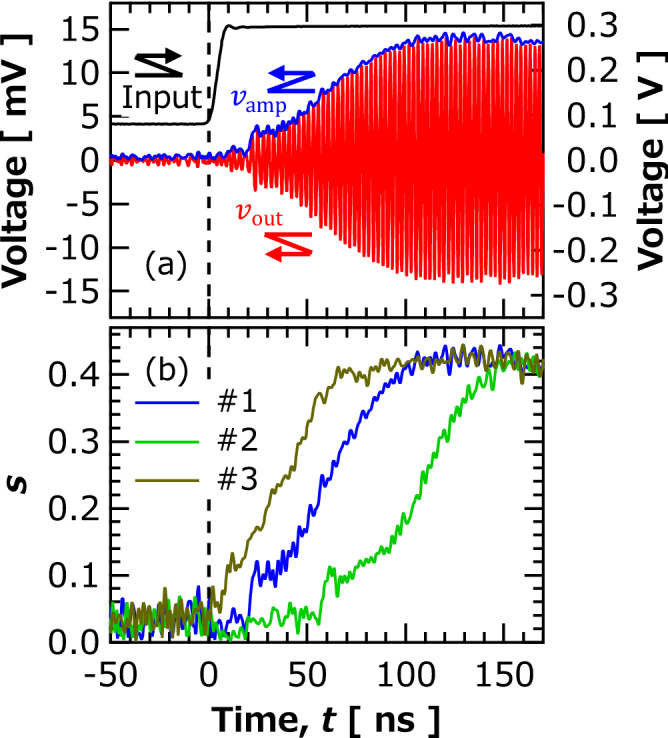
(**a**) Time-domain signal of waveform $v_{{{\text{out}}}} \left( t \right)$ (red), amplitude $v_{{{\text{amp}}}} \left( t \right)$ (blue), and input (black). (**b**) Examples of time evolutions of normalized orbit radius $s$ under same measurement condition. These results were obtained under input condition of $V_{1} = 80$ mV and $V_{2} = 300$ mV.

We prepared a magnetic tunnel junction (MTJ) consisting of FeB free layer, MgO spacer, and CoFeB reference layer. The details of the stacking structure as well as the electric and magnetic properties of MTJ is summarized in “Methods”. The FeB free layer includes a magnetic vortex, where the vortex core is stabilized at the disk center. Applying electric voltage larger than a threshold value, the vortex core starts to move from the disk center and oscillate in the film plane; see also “Methods”. We measured the transient dynamics of the vortex from a non-oscillating to oscillating state by input voltage with two values, $V_{1}$ and $V_{2}$, as shown in Fig. 1. The voltage was switched from $V_{1}$ to another value $V_{2}$ at time $t = 0$ s. The pulse width and rise time of the input voltage used in this study were 1 µs and 6 ns, respectively. The pulse width was determined from the time required to reach a steady oscillating state. The pulse was injected into the STO through a low-pass filter, and the output waveform was measured with an oscilloscope through a high-pass filter. The input and output signals were separated by setting their cutoff frequencies to 100 and 300 MHz, respectively. Figure 2a shows an example of real-time measurements. The input voltage shown with the black line increased from 80 to 300 mV at *t* = 0 s. Note that the initial (final) voltage, 80 (300) mV, is below (above) the threshold voltage; therefore, an auto-oscillation is not excited at $t = 0$ s. The amplitude of the output waveform increased slowly and was saturated after 100 ns passed. We should, however, note that the time evolution of the output voltage does not directly reflect the dynamics of the vortex core. The time evolution of the normalized orbital radius of the magnetic vortex core $s$ can be approximately obtained from the voltage amplitude using^12^,

1 $$ s\left( t \right) = \frac{{4\left( {50 + R\left( t \right)} \right)}}{{50R_{{{\text{parallel}}}} \beta I\left( t \right)MR\left( t \right)}}\left[ {1 - \left( {\frac{{H_{{{\text{op}}}} }}{{H_{{{\text{sat}}}}^{{{\text{ref}}}} }}} \right)^{2} } \right]^{{ - \frac{1}{2}}} \left[ {1 - \left( {\frac{{H_{{{\text{op}}}} }}{{H_{{{\text{sat}}}}^{{{\text{free}}}} }}} \right)^{2} } \right]^{{ - \frac{1}{2}}} v_{{{\text{amp}}}} \left( t \right) , $$

where the voltage amplitude $v_{{{\text{amp}}}} \left( t \right)$, shown with the blue line in Fig. 2a, was “Hilbert envelope”^28^ of the output voltage $v_{{{\text{out}}}} \left( t \right)$ shown with the red line. The notation $R_{{{\text{parallel}}}}$ is the resistance for the parallel magnetic configuration, $H_{{{\text{sat}}}}^{{{\text{free}}}}$ and $H_{{{\text{sat}}}}^{{{\text{ref}}}}$ represent the magnetic fields required to saturate the magnetization in the free layer and reference layer perpendicular to the film plane, respectively, and $\beta$ is the conversion factor, which is fixed to 2/3^29^. Equation (1) relates $v_{{{\text{amp}}}}$, in-plane magnetization components in the free and reference layers $\left[ {1 - \left( {\frac{{H_{{{\text{op}}}} }}{{H_{{{\text{sat}}}}^{{{\text{free}}}} }}} \right)^{2} } \right]^{{ - \frac{1}{2}}}$ and $\left[ {1 - \left( {\frac{{H_{{{\text{op}}}} }}{{H_{{{\text{sat}}}}^{{{\text{ref}}}} }}} \right)^{2} } \right]^{{ - \frac{1}{2}}}$, impedance of the whole circuit, and $s$^12^. The equation is valid when the displacement of the vortex core from the disk radius is relatively small. Using Eq. (1), time evolutions of $s$ with respect to the voltage input can be obtained.

**Figure 3:**
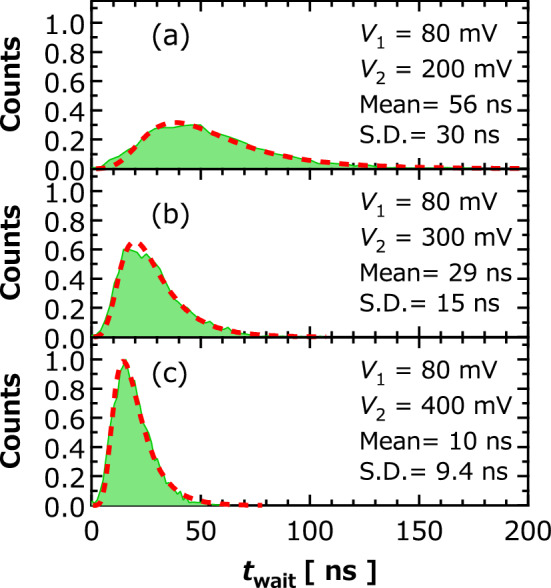
Histograms of waiting time $t_{{{\text{wait}}}}$ obtained under input conditions of $V_{1}$ (**a**) 200, (**b**) 300, and (**c**) 400 mV with $V_{1} = 80$ mV. Mean and standard deviation (S.D.) of $t_{{{\text{wait}}}}$ are also shown. Dotted red lines are fitting curves using Eq. (1). Bin sizes were (a) 4.2, (b) 2.1, and (c) 1.3 ns.

Figure 2b shows examples of the time evolution of $s$ in different trials, where $V_{1}$ and $V_{2}$ are 80 and 300 mV, respectively; see also “Methods” explaining the reason of using finite $V_{1}$. The trials were run with interval more than 1 ms to initialize the non-oscillating state. We can see that the time necessary to move the vortex core from the disk center $\left( {s\sim 0} \right)$ to an oscillating state is different for each trial. For example, the vortex core immediately moved from the disk center in trial 3 (#3) within a few nanoseconds, whereas it stayed close to the disk center for a relatively long time, about 50 ns, in trial 2 (#2). The stochasticity of such a transient response will cause serious issues in neuromorphic computing, where the information of the input time sequence is embedded into the time evolution of the output voltage. Figure 2 also implies that the variation in the rise time from the disk center dominates in the difference of the time evolutions of the vortex-core position. To quantify the distribution of the rising of the vortex-core position, let us define the waiting time $t_{{{\text{wait}}}}$, which is the time required to move the vortex core to $s = 0.1$; see also “Methods” explaining the choice of the standard. Figure 3a–c show histograms of $t_{{{\text{wait}}}}$ obtained by repeating the measurements 5000 times, where $V_{2}$ were (a) 200, (b) 300, and (c) 400 mV. The results indicate that a large input voltage results not only in a fast transition, or equivalently short $t_{{{\text{wait}}}}$, but also in narrowing the distribution of $t_{{{\text{wait}}}}$. This is because spin-transfer effect becomes dominant in transient dynamics from a non-oscillating to oscillating state, thus making the role of stochasticity relatively minor, even though the start of the vortex-core dynamics is a stochastic process.

**Figure 4:**
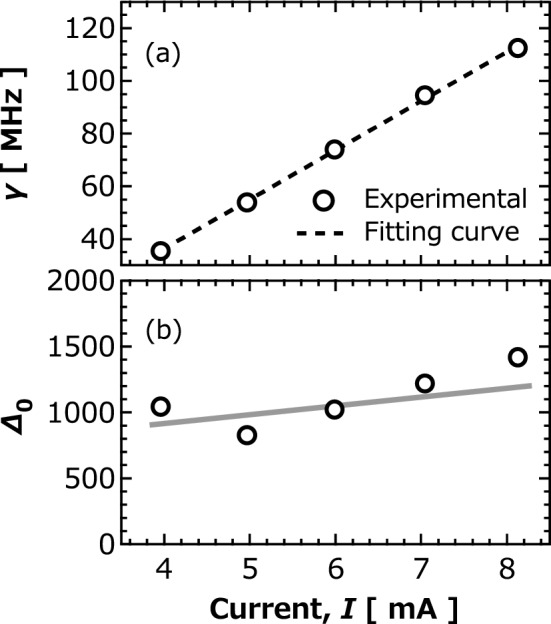
Current dependence of (**a**) $\gamma$ and (**b**) $\Delta_{0}$ obtained from fitting in Fig. 2a-c. Dotted black line is fitting line using ${\upgamma } = 2{\uppi }\alpha f_{{{\text{FMR}}}} \left( {I/I_{{\text{c}}} - 1} \right)$. Solid gray line in (b) is guide for eye.

We had also developed a theoretical analysis on the transient dynamics of the vortex core and found that the distribution function of the vortex core $s$ near the disk center is given by (see also Supplementary Information summarizing the derivation from Fokker–Planck equation)

2 $$ P\left( {s,t} \right) = \frac{2}{\sqrt \pi }\sqrt {\frac{{\gamma \Delta_{0} }}{{\left( {2{\mathcal{D}}\Delta_{0} + \gamma } \right)e^{2\gamma t} - 2{\mathcal{D}}\Delta_{0} }}} {\text{exp}}\left[ { - \frac{{\gamma \Delta_{0} }}{{\left( {2{\mathcal{D}}\Delta_{0} + \gamma } \right)e^{2\gamma t} - 2{\mathcal{D}}\Delta_{0} }}s^{2} } \right] , $$

where ${\Delta }_{0} = \kappa R^{2} \left( {1 - I_{1} /I_{{\text{c}}} } \right)/\left( {2k_{{\text{B}}} T} \right)$ relates to the magnet static energy proportional to $\kappa$, the disk radius $R$, the temperature $T$, the initial current $I_{1}$ corresponding to $V_{1}$, and the threshold current $I_{{\text{c}}}$, whereas ${\upgamma }\sim 2{\uppi }\alpha f_{{{\text{FMR}}}} \left( {I_{2} /I_{{\text{c}}} - 1} \right)$ depends on the damping constant $\alpha$, the ferromagnetic resonance frequency $f_{{{\text{FMR}}}}$, and the input current $I_{2}$ corresponding to $V_{2}$. The histograms of $t_{{{\text{wait}}}}$ in Fig. 3 is given by $P\left( {s = 0.1,t_{{{\text{wait}}}} } \right)$. The dotted lines in Fig. 3a-c correspond to the fitting by Eq. (2), showing a good agreement between experiment and theory. The $\gamma$ and $\Delta_{0}$ obtained by the fitting are summarized in Fig. 4a,b, respectively. As indicated by theoretical results shown above, $\gamma$ increased with the increase in the input current, whereas $\Delta_{0}$ was nearly independent of it. The $I_{{\text{c}}}$ of 2.1 mA estimated from the fitting agrees with the experimental value obtained from the measurement of auto-oscillation.

### Reduction in stochastic behavior by applying radio-frequency signal

Here, we show that the application of an RF signal to the ferromagnet is effective in reducing the stochasticity of the vortex-core dynamics in the time domain. It was shown in Ref.^30^ that a forced synchronization to an RF signal reduces the linewidth of the STO. However, the linewidth only shows the average behavior of the stochastic dynamics, and the role of the RF signal on the vortex-core dynamics in the time domain is still unclear.

**Figure 5:**
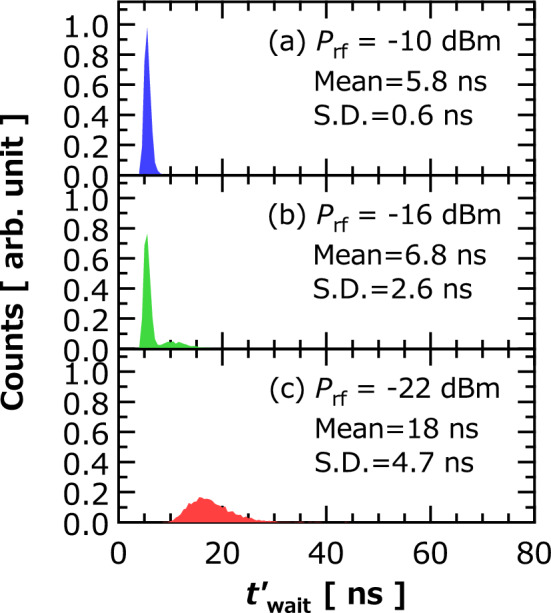
Histograms of waiting time $t^{\prime}_{{{\text{wait}}}}$ obtained under RF input conditions of $P_{{{\text{rf}}}}$ (**a**) − 10 dBm, (**b**) − 16, and (**c**) − 22 dBm. Mean and S.D. of $t_{{{\text{wait}}}}^{\prime }$ are also shown. Input voltage condition was $V_{1} = 0$ mV and $V_{2} = 300$ mV. Bin size was 0.5 ns.

In addition to the input of voltage pulse, we applied an RF magnetic field to the STO, to then measure the vortex dynamics, which followed the same protocol described in previous section. The RF magnetic field was generated by injecting an RF current into a metal line placed above the STO, as shown in Fig. 1. The frequency of the RF signal was set to 350 MHz, which is a peak frequency of thermally excited ferromagnetic resonance. We define waiting time $t_{{{\text{wait}}}}^{^{\prime}}$ as the time required to make $v_{{{\text{amp}}}} \left( t \right)$ half the saturated value; see also “Methods”, where we explain the reason of choosing this standard. Figure 5a–c show the histograms of $t_{{{\text{wait}}}}^{^{\prime}}$ observed for several RF input powers $P_{{{\text{rf}}}}$. A very narrow distribution of $t^{\prime}_{{{\text{wait}}}}$ was obtained at $P_{{{\text{rf}}}} = - 10$ dBm. The narrow shape was maintained by reducing $P_{{{\text{rf}}}}$ to − 16 dBm. As $P_{{{\text{rf}}}}$ further decreased to − 22 dBm, however, the distribution became broad, and the shape of the distribution was similar to that without RF input shown in Fig. 3b. From these results, it was clarified that distribution width of the waiting time is controlled by applying the RF signal.

**Figure 6:**
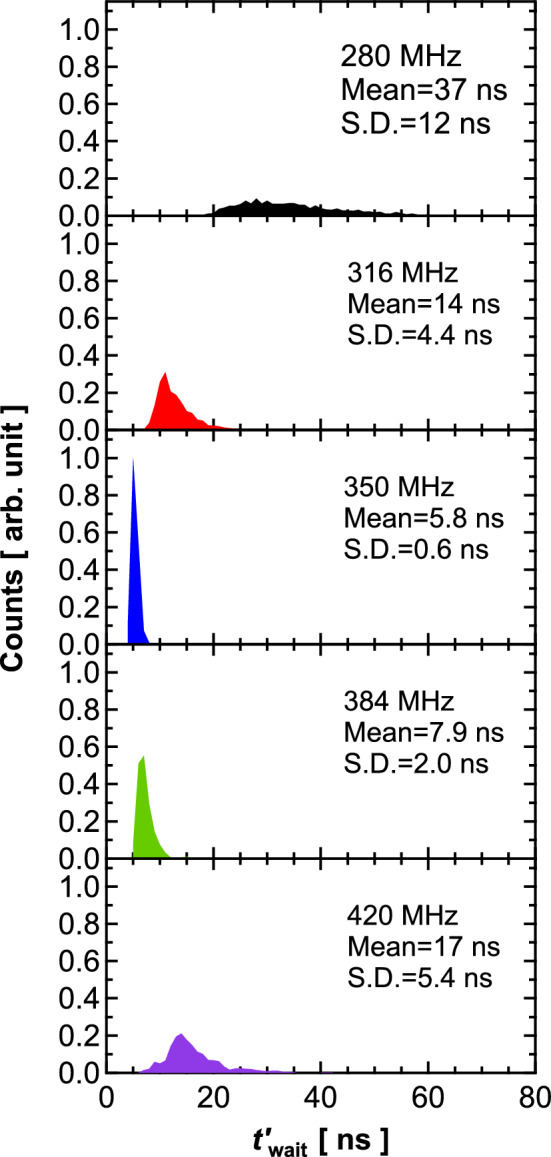
Histograms of $t_{{{\text{wait}}}}^{\prime }$ obtained for various frequencies of RF magnetic field. Mean and S.D. of $t_{{{\text{wait}}}}^{\prime }$ are also shown. RF power was − 10 dBm. Input voltage condition was $V_{1} = 0$ mV and $V_{2} = 300$ mV. Bin size was 1.0 ns.

Note that the reduction in stochasticity in this study differs from that found in forced synchronization^30^. Forced synchronization is a phenomenon in which the oscillation frequency of the vortex core becomes identical to that of the RF signal. We focused on the transient phenomenon in which the instantaneous frequency changes as the vortex core moves from the disk center to a saturated orbital radius, and thus, the frequency is not unique. Our observation is considered to be similar to the resonant phenomena. Figure 6 summarizes the dependence of $t_{{{\text{wait}}}}^{^{\prime}}$ on the frequency of the RF magnetic field. We can see that the distribution is narrow when the frequency is close to the resonance frequency of the vortex core, 350 MHz. The application of the RF magnetic field induces an oscillation of the vortex core near the disk center and suppresses the disturbance of the vortex-core position. Accordingly, the vortex core easily moves from the initial state within a short time and narrow distribution. Microwave-assisted current-induced magnetization instability^31^ is another possible contribution, in which the application of the RF signal results in a reduction in threshold current and leads to fast dynamics of the vortex core. As a result of these effects, stochasticity is relatively suppressed during transient dynamics. In fact, the application of the RF signal in this study resulted not only in narrowing the distribution of $t^{\prime}_{{{\text{wait}}}}$ but also in decreasing the peak position of the histogram, as seen from the comparison between Figs. 3b and 5a,b. While we focused on the time evolution of $s$, recent studies^16,17^ on physical reservoir computing involving STOs showed that the synchronization phenomena exist and are useful when a phase of the dynamical orbit is used for the computation. Regarding the results from this study as well as those from previous studies, applying the RF signal will be an efficient means to control the influence of the stochastic behavior in neuromorphic computing with spintronics technologies whatever the dynamical variables used for computation.

## Conclusion

The transient dynamics from a non-oscillating state to oscillating state of a vortex-type STO via the injection of the voltage pulse was investigated experimentally. It was clarified that the transition response has stochastic waiting time where the oscillation starts randomly with voltage input. It was also found that the distribution width of the waiting time was controlled by applying a radio-frequency magnetic field to the ferromagnet. These findings will open new pathways for the development of the stochastic artificial neuron for neuromorphic computing based on spintronics technologies.

## Methods

### Sample structures

The stacking structure of an MTJ used in this study is Si–O substrate//Ta(10)/Cu(40)/Ta(20)/PtMn(5)/CoFe(2.5)/Ru(0.9)/CoFeB(2.5)/MgO(1)/FeB(5)/MgO(1)/Ta(5)/ Ru(5) (thicknesses in nanometers)^32^. The nominal diameter is 375 nm. The FeB is a free layer, whereas the CoFe/Ru/CoFeB synthetic antiferromagnet is a reference layer. The saturation magnetization and the damping constant of the free layer are 1320 emu/cc and 0.01, respectively. The FeB free layer includes a magnetic vortex due to its relatively large size, whereas the CoFeB reference layer is magnetized along the in-plane easy axis. The tunnel magnetoresistance (TMR) ratio is about 140%. The device size and thickness of the MgO tunnel barrier were designed to have a device resistance of 50 Ω with impedance matching. The actual device resistance is well impedance matched with 48.8–50.3 Ω under the static magnetic field and direct voltage shown below. Auto-oscillation of the magnetic vortex core is excited when the spin-transfer torque via direct electric current compensates the damping torque. Since the spin-transfer torque should have a vertical component to sustain oscillation^33,34^, we applied an out-of-plane magnetic field $H_{{{\text{op}}}}$ of 4.5 kOe to tilt the magnetization in the reference layer. We confirmed that the auto-oscillation is excited for an input voltage larger than 110 mV, corresponding to a threshold current of 2.1 mA.

### Quantities and standards defining waiting time

We evaluated the orbital radius $s$ from the amplitude $v_{{{\text{amp}}}}$ of the output voltage and defined $t_{{{\text{wait}}}}$ as the waiting time necessary to move the vortex core to $s = 0.1$. The initial voltage $V_{1}$ was set to 80 mV. On the other hand, we defined $t_{{{\text{wait}}}}^{^{\prime}}$ as the waiting time necessary to change $v_{{{\text{amp}}}}$ to half the saturated value. The initial voltage was set to 0 mV. The reason we chose these quantities ($s$ and $v_{{{\text{amp}}}}$) and standards ($s = 0.1$ and half the saturated value of $v_{{{\text{amp}}}}$), as well as the different initial voltage, is as follows.

Firstly, it becomes difficult to estimate the vortex-core orbital radius $s$ at the initial time ($t\sim 0$) when $V_{1}$ discussed is zero. This is because Eq. (1) includes the initial current $I\left( t \right)$, which is proportional to *V*_1_; therefore, if $V_{1} = 0$ mV, the estimation of $s$ becomes a divergence. To avoid such a technical issue, we used $V_{1} = 80$ mV in the evaluation of $t_{{{\text{wait}}}}$. Secondly, since the time evolutions of $s$ in different trials indicate that the variation of the saturation time of $s$ mainly originates from that of the rise time from the initial state, we define the waiting time as the time necessary to move the vortex core near the initial state, i.e., we use the standard $s = 0.1$ to define the waiting time $t_{{{\text{wait}}}}$.

**Figure 7:**
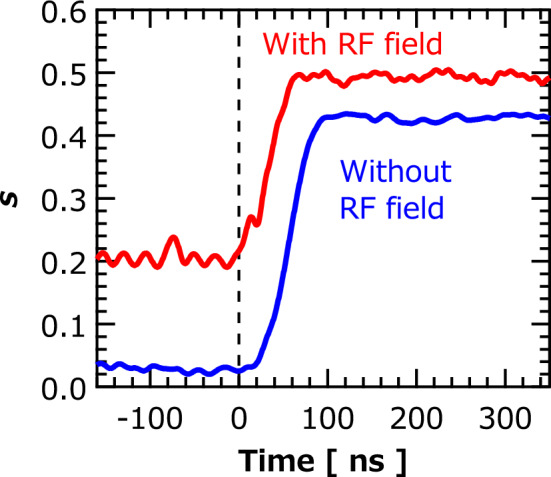
Time evolutions of vortex-core orbital radius *s* in presence and absence of RF magnetic field. Initial voltage was $V_{1} = 80$ mV. Final input voltage was $V_{2} = 300$ mV. RF power was − 10 dBm.

On the other hand, we used $v_{{{\text{amp}}}}$, instead of $s$, to evaluate the waiting time $t_{{{\text{wait}}}}^{^{\prime}}$ due to the following reasons. Firstly, the estimated $s$ often becomes larger than 0.1 in the presence of $V_{1} = 80$ mV because the torque due to the RF magnetic field also moves the vortex core from the disk center; see Fig. 7, where we compare the time evolutions of $s$ in the presence and absence of the RF magnetic field. In this case, the standard $s = 0.1$ is no longer applicable. Another reason to avoid using $s$ relates to the applicability of Eq. (1). As mentioned in Ref.^12^, Eq. (1) is applicable when the vortex core stays near the disk center. Note that an analytical relation, such as Eq. (1), between the position of the vortex core and output voltage can be obtained only when we can specify an alignment of the magnetic moments around the vortex core. For example, Ref.^29^ shows that the numerical factor $\beta = 2/3$ relates the $s$ and $v_{{{\text{amp}}}}$ under two-vortices ansatz, as used in Eq. (1), whereas $\beta = 1$ should be used for a rigid vortex model. Such simplified models are, however, no longer applicable to evaluate $s$ from $v_{{{\text{amp}}}}$ when the vortex core locates far away from the disk center because the deformation of the magnetic moments becomes complicated. Since $s$ in the presence of both the RF magnetic field and spin-transfer torque often becomes large, we used $v_{{{\text{amp}}}}$ instead of $s$ and introduced another standard, i.e., half the saturated value of $v_{{{\text{amp}}}}$, to define the waiting time $t_{{{\text{wait}}}}^{^{\prime}}$.

**Figure 8:**
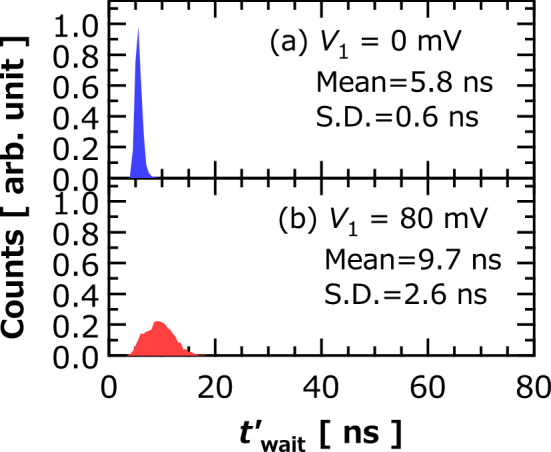
Histograms of $$t_{{{\text{wait}}}}^{\prime }$$ obtained in presence of RF magnetic field. Mean and S.D. of $$t_{{{\text{wait}}}}^{\prime }$$ are also shown. RF power was − 10 dBm. Initial input voltages were (**a**)$$ V_{1} = 0$$ mV and (**b**)$$ V_{1} = 80$$ mV. Bin size was 0.5 ns.

Note that the distribution in the presence of the RF magnetic field becomes narrow when $V_{1}$ is small; see Fig. 8a,b, where we show the distributions of $t_{{{\text{wait}}}}^{^{\prime}}$ under the effect of the RF magnetic field, where $V_{1}$ are (a) 0 and (b) 80 mV. The broadening of the distribution is due to the spin-transfer torque, which tends to move the vortex core from the disk center, whereas the RF magnetic field tends to keep a circular motion with a constant orbital radius. Therefore, the initial distribution in the presence of a finite $V_{1}$ becomes broad compared with that solely formed by the RF magnetic field, resulting in a broad distribution of $t_{{{\text{wait}}}}^{^{\prime}}$. Since the aim of the evaluation of $t_{{{\text{wait}}}}^{^{\prime}}$ is to clarify the control of stochasticity by the RF magnetic field, we use the condition $V_{1} = 0$ mV.

## Supplementary Information

Supplementary Information.
