# Supplementary material for: Control of the stochastic response of magnetization dynamics in spin-torque oscillator through radio-frequency magnetic fields
Source: Sci Rep. 2021 Aug 11;11:16285. doi: 10.1038/s41598-021-95636-w (PMC8357834; doi:10.1038/s41598-021-95636-w)
Supplement: Supplementary file 1 — Supplementary Information. [file 41598_2021_95636_MOESM1_ESM.pdf]

Control of the stochastic response of magnetization dynamics in spin-torque oscillator through radio-frequency magnetic fields

Sumito Tsunegi[1*], Tomohiro Taniguchi[1†], Daiki Suzuki[1], Kay Yakushiji[1], Akio Fukushima[1], Shinji Yuasa[1], and Hitoshi Kubota[1]

[1]National Institute of Advanced Industrial Science and Technology (AIST), Research Center for Emerging Computing Technologies, Tsukuba, Ibaraki 305-8568, Japan.
[*]tsunegi.sb@aist.go.jp
[†]tomohiro-taniguchi@aist.go.jp

ABSTRACT

In this Supplementary Information, we provide theoretical formula of the waiting time derived from the Fokker-Planck equation and used in the analysis of the experiments in the main text.

1 Theoretical analysis on distribution of waiting time

Here, we show that the distribution of the wating time $t_{\rm wait}$ shown in Fig. 3 in the man text is well explained by the solution of the Fokker-Planck equation of the Ornstein-Uhlenbeck process[1–3], which describes the Brownian motion in a medium in the presence of Newtonian frictional force, i.e., the force is proportional to the velocity of the Brownian particle.

1.1 Thiele equation

It has been shown in previous works that the magnetization dynamics in the vortex-type STO is well described by the Thiele equation, which is the equation of motion of the vortex core and agrees well with both experiments and micromagnetic simulations[4–10]. In terms of the vortex core position $\mathbf{X}$ in the film plane measured from the disk center, the Thiele equation is given by

$$-G\hat{\mathbf{e}}_z \times \dot{\mathbf{X}} - D\left(1+\xi s^2\right)\dot{\mathbf{X}} - \frac{\partial W}{\partial \mathbf{X}} + a_J J_{\rm e} p_z \hat{\mathbf{e}}_z \times \mathbf{X} + c a_J J_{\rm e} R_0 p_x \hat{\mathbf{e}}_x = \mathbf{0}, \tag{S.1}$$

where $G = 2\pi pcML/\gamma_0$ and $D = (2\pi\alpha ML/\gamma_0)(1-\mu_D)$ depend on the saturation magnetization M, thickness of the free layer L, gyromagnetic ratio γ_0, Gilbert damping constant α, polarity $p(=\pm 1)$, and chirality $c(=\pm 1)$. A small numerical factor μ_D depends on the model of the magnetic profile, for example, it is $\mu_D = \ln\sqrt{R_0/R}$ in Ref.[6]. The radius of the vortex core is R_0, whereas R is the radius of the ferromagnetic disk. We assume that the magnetization in the reference layer tilts in the xz plane, where $\mathbf{p} = (p_x, 0, p_z)$ is the unit vector pointing in the magnetization direction in the reference layer. The unit vectors in the x and z directions are denoted as $\hat{\mathbf{e}}_x$ and $\hat{\mathbf{e}}_z$, respectively. The magnetic potential energy is[9]

$$W = \frac{\kappa}{2}|\mathbf{X}|^2 + \frac{\kappa'}{4R^2}|\mathbf{X}|^4, \tag{S.2}$$

where $\kappa = (10/9)4\pi M^2 L^2/R$ is used in Ref.[9], whereas a slightly different numerical factor is proposed in Ref.[8]. We introduce the dimensionless parameter $\zeta = \kappa'/\kappa$ for simplicity. The electric current density is $J_{\rm e} = I/(\pi R^2)$ with the current I, whereas $a_J = \pi\hbar P/(2e)$ with the spin polarization P. Nonlinear parameter of the damping is ξ.

We note that, since the size of the vortex core is much smaller than the disk size ($R_0 \ll R$), the last term in Eq. (S.1) is small. Also note that parameter D is proportional to the small constant α. Neglecting the higher order terms of small parameters, the equation of motion for the variable $s = |\mathbf{X}|/R$ becomes[9]

$$\frac{{\rm d}s}{{\rm d}t} = \frac{D\kappa}{G^2}\left[\frac{a_J p_z I G}{\pi R^2 D\kappa} - 1\left(\zeta+\xi\right)s^2\right]s. \tag{S.3}$$

We note that Eq. (S.3) solely depends on s, although the Thiele equation, Eq. (S.1), generally includes two variables. In fact, Eq. (S.1) can be explicitly expressed in terms of s and the phase ψ of the vortex core. However, it has been clarified that the

time evolution of s is approximately independent of the phase[9]. In fact, the numerical solution of Eq. (S.1) shows that the dynamic trajectory of the vortex core is almost a circle[11], indicating that the time evolution of s is nearly independent of ψ. From a different viewpoint, Eq. (S.3) can be regarded as an equation of motion averaged with respect to a fast variable ψ[12,13]. In summary, we can reduce the Thiele equation to an equation of motion for one variable s.

We also note that the right-hand side of Eq. (S.3) includes the first and third order terms of the variable s. Such an equation of motion is called Stuart-Landau equation, which is a standard model of a nonlinear oscillator and was derived to describe turbulence[14]. The solution of the Stuart-Landau equation for a real variable is given in, for example, Ref.[11].

1.2 Thermal activation

Since the experiments have been performed at finite temperature, we should add a random torque due to thermal fluctuation to the Thiele equation. We note that, whereas micromagnetic simulation with the random torque makes the dynamical behavior complex and the calculation cost heavy, the Thiele equation with the random torque[15] well reproduces experimental results[16]. In addition, we will develop the Fokker-Planck theory from the Thiele equation with the random torque, which enables us to discuss the probabilistic behavior due to the thermal fluctuation. The random torque due to the thermal fluctuation is described by random field $\mathbf{h}$, the components of which satisfy the fluctuation-dissipation theorem[17],

$$\langle h_i(\mathbf{r},t)h_j(\mathbf{r}',t')\rangle = \frac{2\alpha k_\mathrm{B}T}{\gamma_0 M}\delta_{ij}\delta(\mathbf{r}-\mathbf{r}')\delta(t-t'). \tag{S.4}$$

Applying the averaging method used in the derivation of the Thiele equation[4], we find that the random torque can be described by random numbers η_s and η_ψ affecting s and ψ of the vortex core position, which satisfy

$$\langle \eta_i(t)\eta_j(t')\rangle = 2Dk_\mathrm{B}T\delta(t-t'). \tag{S.5}$$

Adding the random torque to the Thiele equation, the equation of motion for s, Eq. (S.3), becomes,

$$\frac{\mathrm{d}s}{\mathrm{d}t} = \frac{D\kappa}{G^2}\left[\frac{a_J p_z I G}{\pi R^2 D\kappa} - 1\,(\xi+\zeta)\,s^2\right]s - \frac{\eta_s}{GR}. \tag{S.6}$$

Equation (S.6) is the Langevin equation for the variable s.

1.3 Fokker-Planck equation

As mentioned above, the Thiele equation consists of the equations of motion for the variable s and the phase ψ, and the equation for s is approximately independent from the phase. We also note that the angular velocity of the vortex-core oscillation is also approximately independent of the phase. Accordingly, the probability density $P(s,\psi)$ to find the vortex core in a certain infinitesimal area $\mathrm{d}s\mathrm{d}\psi$ on a disk solely depends on the variable s. In addition, we focus on the vortex-core dynamics close to the disk center, where $s \ll 1$. Applying a standard method[2,3,13] to derive the Fokker-Planck equation from the Langevin equation, we find that the Fokker-Planck equation for the probability density P of s is then given by

$$\frac{\partial P}{\partial t} + \frac{\partial J}{\partial s} = 0, \tag{S.7}$$

where the probability flux density J is

$$J(s,t) = \gamma s P - \mathscr{D}\frac{\partial P}{\partial s}. \tag{S.8}$$

Here $\gamma = (D\kappa/G^2)[(a_J p_z I G)/(\pi R^2 D\kappa) - 1] \sim 2\pi\alpha f_\mathrm{FMR}(I/I_\mathrm{c} - 1)$ is the damping rate, whereas $\mathscr{D} = k_\mathrm{B}TD/(G^2R^2)$ is the diffusion constant at temperature T, $f_\mathrm{FMR} = \kappa/(2\pi G)$ is the ferromagnetic resonance (FMR) frequency when the vortex core locates at the disk center, and $I_\mathrm{c} = \pi R^2 D\kappa/(a_J p_z G)$ is the threshold current to move the vortex core from the disk center. The higher order terms of s in Eq. (S.6) is neglected in Eq. (S.8) because of the assumption $s \ll 1$. We note that Eq. (S.7) with Eq. (S.8) has the same form of the Fokker-Planck equation of the Ornstein-Uhlenbeck process[1]. The Uhlenbeck and Ornstein studied the Brownian motion in a medium in the presence of Newtonian frictional force, and found that the integral kernel of the process is given by

$$\mathscr{G}(s,s_0,t) = \sqrt{\frac{\gamma}{2\pi\mathscr{D}\left(\mathrm{e}^{2\gamma t}-1\right)}}\exp\left[-\frac{\gamma\left(s\mathrm{e}^{-\gamma t}-s_0\right)^2}{2\mathscr{D}\left(1-\mathrm{e}^{-2\gamma t}\right)}\right]. \tag{S.9}$$

Note that the original work of Uhlenbeck and Ornstein used the friction coefficient $\beta = -\gamma$, but we use negative friction coefficient γ because we are interested in an excitation of the auto-oscillation of the magnetic vortex core where the spin-transfer torque overcomes the damping torque. It should also be noted that the variable s of the present problem is restricted in the range of $0 \leq s \leq 1$ because it represents the distance of the vortex core measured from the disk center normalized by the disk radius R. Therefore, the probability flux density should satisfy $J = 0$ at $s = 0$. The boundary condition implies $\partial P/\partial s = 0$ at $s = 0$. In addition, the probability density should be zero in the limit of $s \to \infty$, where, since we focus on the vortex-core dynamics near the disk center ($s \ll 1$), the boundary condition at the edge of the disk ($s = R$) can be replaced with that at $s \to \infty$. Regarding these boundary conditions, the solution of the Fokker-Planck equation is given by

$$P(s,t) = \sqrt{\frac{\gamma}{2\pi\mathscr{D}\left(e^{2\gamma t}-1\right)}}\int_0^\infty P(s_0,0)\left\{\exp\left[-\frac{\gamma\left(se^{-\gamma t}-s_0\right)^2}{2\mathscr{D}\left(1-e^{-2\gamma t}\right)}\right]+\exp\left[-\frac{\gamma\left(se^{-\gamma t}+s_0\right)^2}{2\mathscr{D}\left(1-e^{-2\gamma t}\right)}\right]\right\}ds_0. \tag{S.10}$$

We note that Eq. (S.10) has two integral kernels, where one is given by Eq. (S.9) whereas the other is obtained from Eq. (S.9) by changing the sign of s. Accordingly, the boundary condition at $s = 0$ mentioned above and the conservation of the probability density in the region of $0 \leq s$ are satisfied. Assuming that the initial distribution $P(s_0, 0)$ is given by the Boltzmann distribution function $P(s,0) = 2\sqrt{\Delta_0/\pi}e^{-\Delta_0 s^2}$, Eq. (S.10) becomes

$$P(s,t) = \frac{2}{\sqrt{\pi}}\sqrt{\frac{\gamma\Delta_0}{\left(2\mathscr{D}\Delta_0+\gamma\right)e^{2\gamma t}-2\mathscr{D}\Delta_0}}\exp\left[-\frac{\gamma\Delta_0}{\left(2\mathscr{D}\Delta_0+\gamma\right)e^{2\gamma t}-2\mathscr{D}\Delta_0}s^2\right], \tag{S.11}$$

where $\Delta_0 = \kappa R^2(1 - I_1/I_c)/(2k_B T)$ is the effective thermal stability determining the initial condition of the distribution function. A factor $(1 - I_1/I_c)$ with the current I_1 corresponding to V_1 comes from the steady-state solution of the Fokker-Planck equation and describes the deformation of the equilibrium distribution function due to spin-transfer torque. We note that the distribution of the waiting time studied in the main text is given as $P(s = 0.1, t_{wait})$.

Let us briefly discuss the properties of Eq. (S.11). The distribution of the waiting time is maximized at

$$t = \frac{1}{\gamma}\ln\sqrt{\frac{2\mathscr{D}\Delta_0+2\gamma\Delta_0 s^2}{2\mathscr{D}\Delta_0+\gamma}}. \tag{S.12}$$

Note that the distribution function satisfies the following translational symmetry,

$$P(s,t,\Delta_0) = P(s,t+u,\Delta'), \tag{S.13}$$

where u is given by

$$u = -t + \frac{1}{\gamma}\ln\left[\sqrt{\frac{2\Delta'}{2\mathscr{D}\Delta'+\gamma}}\sqrt{\frac{\mathscr{D}\operatorname{plog}\left(Qe^Q\right)-\gamma s^2}{\operatorname{plog}\left(Qe^Q\right)}}\right], \tag{S.14}$$

where plog is the product logarithm (Lambert W function), whereas Q is given by

$$Q = \frac{-2\gamma\Delta_0 s^2}{\left(2\mathscr{D}\Delta_0+\gamma\right)e^{2\gamma t}-2\mathscr{D}\Delta_0}. \tag{S.15}$$

The translational symmetry given by Eq. (S.13) indicates that the shape of the histogram of the waiting time remains unchanged even if the thermal stability is changed, although the peak position shifts by factor u. In a magnetic system, however, thermal stability is mainly determined by the magnetic potential energy characterized by κ; therefore, the shape of the histogram will change by changing the thermal stability because κ affects not only thermal stability but also other parameters such as the threshold current.

References

1. Uhlenbeck, G. E. & Ornstein, L. S. On the theory of the Brownian motion. *Phys. Rev.* **36**, 823 (1930).
2. Risken, H. *The Fokker-Planck Equation* (Springer, Berlin, 1989).
3. Coffey, W. T., Kalmykov, Y. P. & Waldron, J. T. *Langevin Equations with Applications in Physics, Chemistry and Electrical Engineering* (World Scientific Publishing, Singapore, 1996).

4. Thiele, A. A. Steady-State Motion of Magnetic Domain. *Phys. Rev. Lett.* **30**, 230 (1973).
5. Guslienko, K. Y., Han, X. F., Keavney, D. J., Divan, R. & Bader, S. D. Mangetic Vortex Core Dynamics in Cylindrical Ferromagnetic Dots. *Phys. Rev. Lett.* **96**, 067205 (2006).
6. Guslienko, K. Y. Low-frequency vortex dynamics susceptibility and relaxation in mesoscopic ferromagnetic dots. *Appl. Phys. Lett.* **89**, 022510 (2006).
7. Khvalkovskiy, A. V., Grollier, J., Dussaux, A., Zvezdin, K. A. & Cros, V. Vortex oscillations induced by spin-polarized current in a magnetic nanopillar: Analytical versus micromagnetic calculations. *Phys. Rev. B* **80**, 140401(R) (2009).
8. Goto, M. *et al.* Electric spectroscopy of vortex states and dynamics in magnetic disks. *Phys. Rev. B* **84**, 0644046 (2011).
9. Dussaux, A. *et al.* Field dependence of spin-transfer-induced vortex dynamics in the nonlinear regime. *Phys. Rev. B* **86**, 014402 (2012).
10. Grimaldi, E. *et al.* Response to noise of a vortex based spin transfer nano-oscillator. *Phys. Rev. B* **89**, 104404 (2014).
11. Yamaguchi, T. *et al.* Step-like dependence of memory function on pulse width in spintronics reservoir computing. *Sci. Rep.* **10**, 19536 (2020).
12. Hillebrands, B. & Thiaville, A. (eds.) *Spin Dynamics in Confined Magnetic Structures III* (Springer, Berlin, 2006).
13. Bertotti, G., Mayergoyz, I. & Serpico, C. *Nonlinear Magnetization Dynamics in Nanosystems* (Elsevier, Oxford, 2009).
14. Kuramoto, Y. *Chemical Oscillations, Waves, and Turbulence* (Dover, 2003).
15. Khalsa, G., Stiles, M. D. & Grollier, J. Critical current and linewidth reduction in spin-torque nano-oscillators by delayed self-injection. *Appl. Phys. Lett.* **106**, 242402 (2015).
16. Tsunegi, S. *et al.* Self-Injection Locking of a Vortex Spin Torque Oscillator by Delayed Feedback. *Sci. Rep.* **6**, 26849 (2016).
17. Brown Jr, W. F. Thermal Fluctuations of a Single-Domain Particle. *Phys. Rev.* **130**, 1677 (1963).
